# Atlanta Academy of Medicine

**Published:** 1878-12

**Authors:** J. G. Westmoreland

**Affiliations:** Reporter


### Reports of Societies.

### ATLANTA ACADEMY OF MEDICINE.

DR. J. G. WESTMORELAND, REPORTER.

ATLANTA, GA., Nov. 11, 1878.

Dr. J. F. Alexander, President, in the chair.

Dr. A. W. Calhoun reported two cases of cleft palate, both of which extended only through the soft parts. Nothing unusual occurred in the cases, both having recovered readily, except that a small point failed to unite in one of them, which, however, seems to improve by the application of nitrate of silver. He desires the opinion of those having experience in such operations, as to the best mode of relieving such difficulties.

Dr. J. T. Johnson reported a case of femoral hernia requiring an operation for the relief of strangulation. The tumor was opened and the supposed stricture at Gimbernatt's ligament was well cut, but without giving freedom sufficient to return the strangulated bowel. The sack was then opened, and at the mouth of the same the stricture was found and divided; after which the bowel was readily returned. The case had nothing unusual except the point of stricture being located at the sac instead of the femoral ring, and also the comparative rarity of this form of hernia.

November 18, 1878.

Dr. J. F. Alexander, President, in the chair.

Dr. H. L. Wilson reported a case of chancroid ulcer of the penis, which had destroyed the glands and a portion of the prepuce. The case had received bad treatment, or perhaps no treatment at all, previous to consulting Dr. Wilson, and it seems from the appearance when presented that the ulceration of the glands had gone on with the prepuce contracted in front of it, so that the process of ulceration was not probably detected by the patient or any one who may have prescribed for him, till ulceration through the front or upper portion of the foreskin had revealed the destructive process which had been going on within. The remnant of prepuce was so arranged by Dr. W. that a kind of a make-shift of a glands was formed.

Dr. J. H. Low reported a case of uterine disease in a lady forty-three years of age. The disease has existed for fifteen years, and has confined her to her room for four years. From extensive and minute digital examination, the conclusion he has arrived at is that stricture of the internal os exists as the main cause of trouble. He had first formed this opinion from detection of the contracted portion by the finger, and from the reported pain in the discharge of blood, which is copious most of the time. The patient complains of burning sensation of the surface of right side of the body and lower extremity, with severe pain in the limbs. He desires the opinion of members on this case.

Dr. J. T. Johnson thought the examination insufficient to determine the course that should be pursued.

Dr. J. G. Westmoreland said the nervous sensations described were common in any serious disease of the uterus. Pain and various strange sensations are the result of reflex nervous disturbance. He thinks more thorough examination is necessary to determine even the state of the cervical canal and os internum, which cannot be done satisfactorally by digital exploration. Evidently the intra-uterine surface is the subject of disease, and its nature should be ascertained so that proper applications for its relief can be made.

Dr. J. T. Johnson reported two cases of syphilitic chancre. There was nothing very unusual in the cases, except as to the period of incubation. Both were contracted from the same source, and while in one the chancre was developed in five weeks, in the other nine weeks elapsed before the local manifestations appeared. He expressed confidence in the correctness of the history which fixes the time of incubation, and the character of the chancre is, he thinks, undoubted. He had read of three weeks, and even forty or fifty days, but sixty-three days, as was found to be the period of incubation of one case, is a longer time than he has seen recorded.

Dr. Baird reported progress of so-called idiopathic tetanus. The case has gradually improved since last report. There is still irregularity of movement, but no decided tetanic spasm. A kind of delirium is occasionally present, particularly on waking from sleep. He is now taking eight drops every six hours of tincture gelsemium.

Dr. Calhoun reported progress in the case of cystocircus reported some months ago. The eye thus affected has still less vision, and of course will entirely fail ultimately. He also reported two cases of bony tumor of the meatus auditorius. The tumor, commencing on the posterior wall, gradually filled up the canal. Hearing in the ear affected is almost entirely lost. He proposes relief by drilling off the oseous tumor by a peculiar instrument for the purpose.

Dr. J. F. Alexander, the President, reported a case of twins, in which one of the children was found dead, and had evidently been in a state of decomposition for several days. The mother had miscarried several times, the fœtus being dead. Syphilitic taint was traced, and some years ago treatment was given to relieve the offspring of the father, who was contaminated. It was instituted by giving the mother iodine, and hence the full time for this delivery. He does not think the death of one of the children depended on the syphilis, as heretofore. He thinks the mother had not been contaminated, but that the taint proving destructive to the children emanated entirely from the father.

Some discussion was had on the question whether this taint could be communicated to the fœtus without infecting the mother. Also in regard to the beneficial effects of iodide potassium in inherited syphilis. Dr. Johnson thought it is not, while Dr. Baird believed it is.

Dr. Connally, in connection with this subject, mentioned a case of inherited syphilis from the father, and a child born afterwards was healthy. The mother was the subject of uterine disease after the birth of the last child, which disappeared under syphilitic treatment. He thinks the syphilitic taint thus proven in the mother was communicated by the father through the fœtus to the mother.

